# Supplementary material for: Local expectation violations result in global activity gain in primary visual cortex
Source: Sci Rep. 2016 Nov 22;6:37706. doi: 10.1038/srep37706 (PMC5118700; doi:10.1038/srep37706)
Supplement: Supplementary Information [file srep37706-s1.pdf]

# Local expectation violations result in global activity gain in primary visual cortex

Peter Kok[1,2,*], Lieke L.F. van Lieshout[1] and Floris P. de Lange[1]

1. Radboud University Nijmegen, Donders Institute for Brain, Cognition and Behaviour, Kapittelweg 29, 6525 EN Nijmegen, The Netherlands
2. Princeton University, Princeton Neuroscience Institute, 301 Peretsman-Scully Hall, Princeton, NJ 08544

***Corresponding author:**

Peter Kok

Princeton Neuroscience Institute

Princeton University

301 Peretsman-Scully Hall, Princeton, NJ 08544

Phone: 1 609 258 8729

E-mail: pkok@princeton.edu

**Supplementary information**

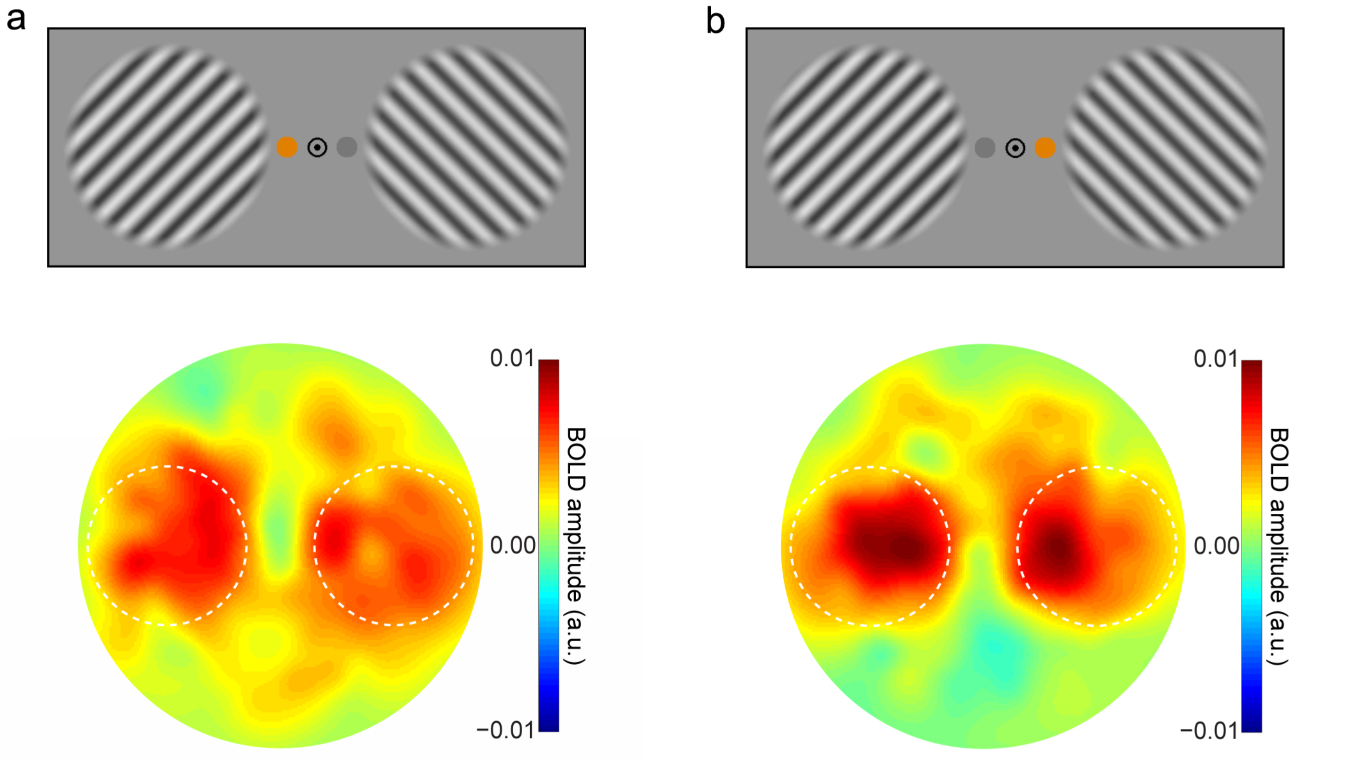


**Supplementary Figure S1. Retinotopic reconstruction of expectation effect per hemifield.** That is, ‘Cued grating Unexpected – Expected’, separately for when the cued grating was on the left (**a**) and when it was on the right (**b**). It can clearly be seen that the expectation effect is bilateral in both cases. This is the same effect as shown in Figure 2C, in which results were collapsed over ‘cued left’ and ‘cue right’ trials.

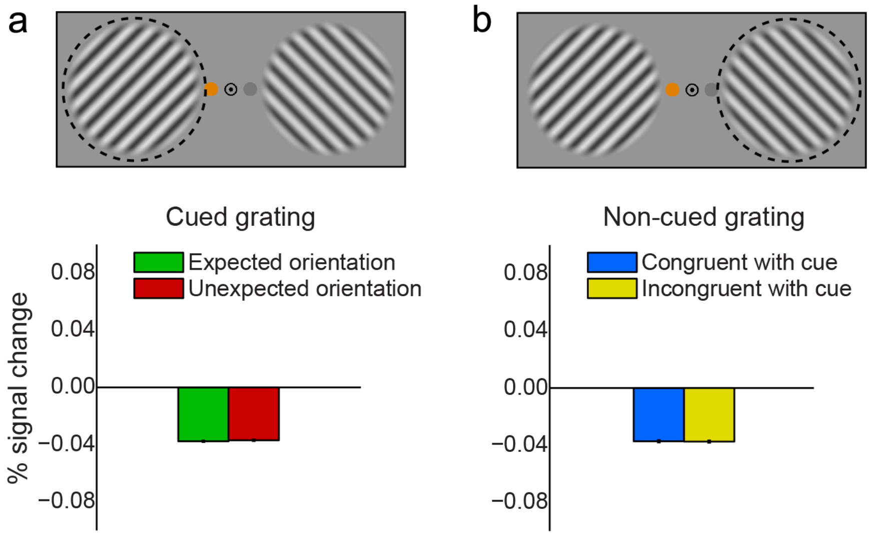


**Supplementary Figure S2. BOLD amplitude in non-stimulated background.** (**a**) Here, trials are split up according to whether the gratings on the cued side had the expected or unexpected orientation. BOLD amplitude is shown for the cortical 'background' ROI, i.e. voxels with a pRF that overlapped with neither of the two gratings. (**b**) Here, trials are split up according to whether the orientation of the gratings on the non-cued side was congruent or incongruent with the expectation cue. Same cortical ROI as in (A). Error bars indicate within-subject SEM.

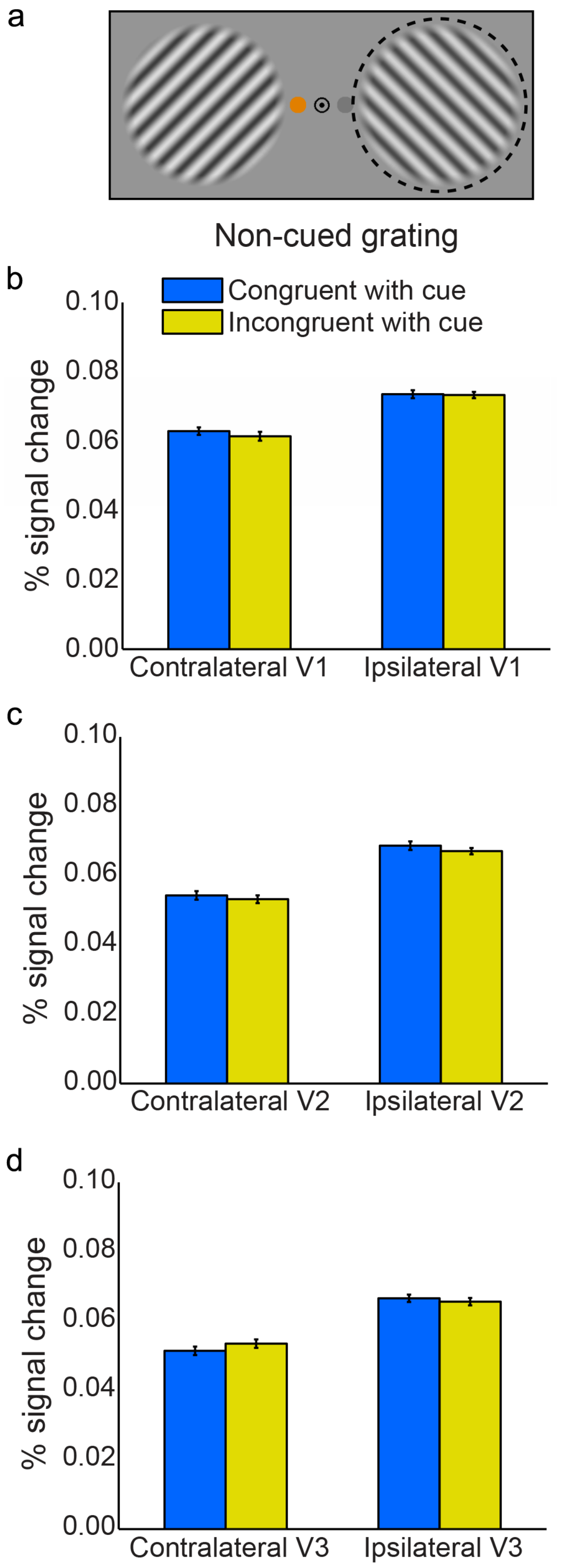


**Supplementary Figure S3. ROI BOLD amplitude evoked by non-cued gratings.** (**a**) Here, trials are split up according to whether the orientation of the gratings on the non-cued side was congruent or incongruent with the expectation cue. Note that the orientation of these gratings was independent of the expectation cue, so no effect would be expected if feature-based expectation is specific to the cued grating. BOLD amplitude is shown separately for the cortical ROI in which the non-cued grating was processed (i.e. contralateral hemisphere) as well as for the ROI in the opposite (i.e. ipsilateral) hemisphere. Results are shown separately for (**b**) V1, (**c**) V2, and (**d**) V3. Note that the BOLD response is lower contralateral to the non-cued grating than contralateral to the cued grating (i.e., ipsilateral to the non-cued grating), as a result of spatial attention. There was no difference in BOLD response depending on whether the orientation of non-cued gratings was congruent (blue bars) or incongruent (yellow bars) with the expectation cue. Error bars indicate within-subject SEM. (See Figure 3 for BOLD amplitude conditioned on whether the cued grating matched or violated the expectation cue.)
